# Prediction of Successful Ovarian Protection Using Gonadotropin-Releasing Hormone Agonists During Chemotherapy in Young Estrogen Receptor-Negative Breast Cancer Patients

**DOI:** 10.3389/fonc.2020.00863

**Published:** 2020-06-16

**Authors:** Dong-Yun Lee, Ji-Yeon Kim, Jonghan Yu, Seok Won Kim

**Affiliations:** ^1^Department of Obstetrics and Gynecology, Samsung Medical Center, Sungkyunkwan University School of Medicine, Seoul, South Korea; ^2^Department of Medicine, Samsung Medical Center, Sungkyunkwan University School of Medicine, Seoul, South Korea; ^3^Department of Surgery, Samsung Medical Center, Sungkyunkwan University School of Medicine, Seoul, South Korea

**Keywords:** breast cancer, chemotherapy, ovarian protection, gonadotropin-releasing hormone, anti-Müllerian hormone

## Abstract

**Background:** It is important to identify factors predicting successful ovarian protection using gonadotropin-releasing hormone (GnRH) agonists during chemotherapy. However, only a few studies have prospectively assessed this issue in young breast cancer patients.

**Objective:** This study evaluated the predictive factors for successful ovarian protection with GnRH agonists during chemotherapy in young estrogen receptor-negative breast cancer patients.

**Materials and Methods:** This prospective study analyzed 67 estrogen receptor-negative breast cancer patients ≤40 years of age who were longitudinally assessed after receiving GnRH agonists during cyclophosphamide-based chemotherapy for ovarian protection. Associations between clinical characteristics or pretreatment hormones and successful ovarian protection [resumption of menstruation and anti-Müllerian hormone (AMH) ≥1 ng/ml].

**Results:** The mean age and pretreatment serum level of AMH were 33.2 years and 4.57 ng/ml, respectively. At 12 months after the completion of chemotherapy, most women (97%) experienced the resumption of menstruation. However, the proportion of patients with AMH ≥1 ng/ml at 12 months was 70.1%. In multivariate analyses, only the pretreatment serum AMH level (*P* < 0.001) was predictive for AMH ≥1 ng/ml at 12 months. Receiver operating characteristic curve analyses of pretreatment AMH exhibited an area under the curve of 0.866 (95% CI = 0.777–0.955) for AMH ≥1 ng/ml at 12 months. The cutoff value for the prediction of AMH concentration ≥1 ng/ml at 12 months was 2.87 ng/ml of pretreatment AMH with a sensitivity of 0.87 and a specificity of 0.75.

**Conclusions:** Pretreatment AMH (2.87 ng/ml) is a useful predictor for AMH ≥1 ng/ml at 12 months after receiving GnRH agonists in young estrogen receptor-negative breast cancer patients. This finding can help improve decision-making regarding fertility preservation.

## Introduction

Breast cancer is a commonly diagnosed malignancy in young premenopausal women. Since the prognosis has been improved over the decades (1), the quality of life in young survivors is becoming a more important issue and deserves high interest and priority in the consequences of breast cancer treatment (2).

Considering its more aggressive nature (3), chemotherapy is often required in young breast cancer patients. It is well-known that alkylating agents have a high risk of gonadotoxicity and may result in long-term sequelae such as early menopause (4). Therefore, health care providers should consider and provide fertility preservation options to young cancer patients (5).

Currently, cryopreservation is the only established and standard method for fertility preservation in young women with breast cancer (6, 7). However, accumulated evidence now supports the efficacy of ovarian protection using gonadotropin-releasing hormone (GnRH) agonists during chemotherapy to prevent chemotherapy-related amenorrhea or early menopause and also to preserve fertility for future pregnancies (8–12).

However, as introduced in recent guidelines (7), GnRH agonists may reduce the risk of early menopause (or loss of menstruation), but it is not a proven fertility preservation method. Serum anti-Müllerian hormone (AMH) levels indeed decreased after ovarian protection with GnRH agonists (10). Since not all women can experience the beneficial effects of GnRH agonists for preserving fertility, it is important to identify factors predicting successful ovarian protection. Unfortunately, only a few studies have prospectively assessed this issue (10, 13). A better prediction can be helpful for selecting good candidates for ovarian protection with GnRH agonists in young breast cancer patients.

The aim of this study was to address the predictive factors for successful ovarian protection with GnRH agonists during cyclophosphamide-based chemotherapy in young breast cancer patients.

## Materials and Methods

### Study Population

This prospective study included all women with estrogen receptor-negative breast cancer stages I–III who were ≤40 years old and received GnRH agonists for ovarian protection during chemotherapy at Young Breast Cancer Clinic at the Samsung Medical Center in Seoul, Korea, from January 2013 to December 2017. Our cohort was already introduced elsewhere (13).

Women were excluded from this study if they (1) did not have regular menstruation at the baseline; (2) had serum AMH levels less than 1 ng/ml before treatment; (3) had a previous history of any gonadotoxic treatment such as chemotherapy, radiation, or surgery; (4) used any hormonal contraceptives that could affect serum AMH levels; and (5) had a short follow-up duration (less than 12 months).

The study protocol was approved by the Institutional Review Board of the Samsung Medical Center, and informed consent was obtained from each patient.

### Treatment

One of the following three regimens were determined for chemotherapy based on the clinical judgment by medical oncologists: doxorubicin (60 mg/m^2^) and cyclophosphamide (600 mg/m^2^) for four cycles with or without four additional cycles of paclitaxel (175 mg/m^2^) or cyclophosphamide (500 mg/m^2^), doxorubicin (50 mg/m^2^), and 5-fluorouracil (500 mg/m^2^) for six cycles. The patients were treated with each regimen every 3 weeks.

Before starting chemotherapy, the patients received an injection of GnRH agonists regardless of their menstrual cycle and thereafter every 4 weeks until the completion of chemotherapy.

### Measurements

Menstrual history was assessed before and after treatment. Serum follicle-stimulating hormone (FSH) and AMH were checked before and 12 months after chemotherapy. The serum level of AMH was measured using Gen II ELISA (Beckman Coulter, Fullerton, CA, USA) according to the manufacturer's instructions. The minimum detectable concentration was 0.16 ng/ml, and the inter- and intra-assay coefficients of variation were 5.6 and 5.4%, respectively. Serum FSH concentration was measured using a radioimmunoassay. Blood tests were performed during visits to the clinic, regardless of menstrual phase.

Ovarian function was assessed based on menstruation and serum AMH levels, and both the resumption of menstruation and AMH ≥1 ng/ml at 12 months after chemotherapy were considered factors of successful ovarian protection in the present study. We set the cutoff value of serum AMH level considering the criteria for poor ovarian response (14, 15) and previous studies predicting future fertility potential (16–18).

### Statistical Analysis

Clinical characteristics and hormone profiles were compared according to the serum AMH levels at 12 months after the end of chemotherapy. Data are presented as the mean ± standard deviation or number (percentage). The Mann–Whitney or *t*-test was used to compare continuous variables, and chi-square or Fisher's exact test was used to compare categorical data.

Simple and multiple logistic regressions were used to test the association between successful ovarian protection and its related factors. Potential confounders that might affect ovarian function after chemotherapy such as age, body mass index, baseline FSH and AMH, cyclophosphamide dose, and days from GnRH agonist administration to chemotherapy were included in the multivariate analysis.

The area under the curve underwent receiver operating characteristic curve analysis for serum AMH concentration ≥1 ng/ml at 12 months. Pretreatment AMH was used as a predictor. Model validation (estimate of the mean and 95% CI) was executed through 1,000 bootstrap resamples for *R*-square, the area under the curve, and the concordance index.

All *P*-values were two-tailed, and *P* < 0.05 was considered to be statistically significant. SAS version 9.4 (SAS Institute Inc., Cary, NC, USA) and R 3.6.0 (Vienna, Austria; http://www.R-project.org/) were used for analyses.

## Results

**Figure 1:**
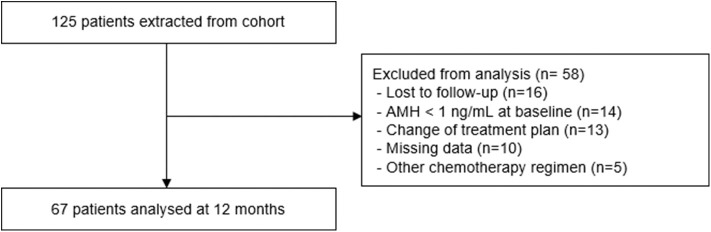
Flow diagram for the selection of the study subjects.

During the 5-year study period, a total of 125 young women with estrogen receptor-negative breast cancer received GnRH agonists for ovarian protection during chemotherapy. Among them, 14 women exhibited a low serum level of AMH before treatment, while 16 were lost to follow-up before 12 months. Finally, a total of 67 women were included for analyses (Figure 1).

Table 1 presents the clinical characteristics of the study subjects. The mean age was 33.2 years. The most commonly used chemotherapy regimen was doxorubicin and cyclophosphamide followed by taxane. The mean cumulative dose of cyclophosphamide was 3,841.9 ± 432.2 mg, and the mean number of days from the first GnRH agonist injection to the first chemotherapy session was 7.9 ± 8.3 days. The mean pretreatment serum level of AMH was 4.6 ± 2.5 ng/ml.

**Table 1:** Characteristics of the study subjects.

**Characteristics**	***N* = 67**
Age (years)	33.2 ± 3.6
Body mass index (kg/m^2^)	21.6 ± 3.0
Age at menarche (years)	12.7 ± 1.3
Parity (*n*)	0.7 ± 0.8
Married	36 (53.7%)
**Stage**	
I	23 (34.3%)
II	27 (40.3%)
III	17 (25.4%)
HER2 positive	16 (23.9%)
**Type of surgery**	
BCS	24 (35.8%)
PM	28 (41.8%)
MRM	5 (7.5%)
TM	10 (14.9%)
**Chemotherapy regimen**	
AC	5 (7.5%)
AC followed by taxane	51 (76.1%)
CAF	11 (16.4%)
Cumulative cyclophosphamide dose (g)	3,841.9 ± 432.2
**Type of GnRH agonist**	
Goserelin	55 (82.1%)
Leuplin	12 (17.9%)
Days from GnRH agonist administration to chemotherapy (*n*)	7.9 ± 8.3
Baseline FSH	4.5 ± 2.6
Baseline AMH	4.57 ± 2.54

*Data are presented as mean ± SD or n (percentage)*.

*AC, doxorubicin and cyclophosphamide; AMH, anti-Müllerian hormone; BCS, breast-conserving surgery; CAF, cyclophosphamide, doxorubicin, and 5-fluorouracil; FSH, follicle-stimulating hormone; GnRH, gonadotropin-releasing hormone; HER2, human epidermal growth factor receptor 2; MRM, modified radical mastectomy; PM, partial mastectomy; TM, total mastectomy*.

At 12 months after the end of chemotherapy, most patients (97%, 65/67) had experienced the resumption of menstruation, although not all of them were regular. However, the serum levels of AMH significantly decreased compared to the baseline (from 4.6 to 1.6 ng/ml; *P* < 0.001), and 47 patients (70.1%) had serum AMH ≥1 ng/ml at 12 months. The mean values of AMH in patients with AMH <1 ng/ml and those with AMH ≥1 ng/ml were 0.3 ± 0.2 and 2.2 ± 1.6 ng/ml, respectively. In addition, the mean difference in serum AMH levels between the baseline and at 12 months was −2.1 ± 1.5 ng/ml in patients with AMH <1 ng/ml and−3.3 ± 2.3 ng/ml in those with AMH ≥ 1 ng/ml at 12 months, which did not reach a statistical significance between the two groups (data not shown).

Table 2 shows the comparison of the characteristics of the study subjects according to their serum levels of AMH at 12 months. Pretreatment serum AMH levels were significantly different between failure (AMH <1) and successful ovarian protection (AMH ≥1) (2.5 ± 1.5 vs. 5.5 ± 2.4 ng/ml; *P* < 0.001). However, age, dose of cyclophosphamide, days from the first GnRH agonist injection to chemotherapy, and pretreatment FSH did not differ. When multivariate analysis was performed, only AMH was associated with a greater likelihood of serum AMH level ≥1 ng/ml at 12 months (adjusted odds ratio = 2.193, 95% CI = 1.456–3.303; *P* < 0.001).

**Table 2:** Pretreatment characteristics by serum level of AMH at 12 months.

**Variables**	**AMH <1 ng/ml (*n* = 20)**	**AMH ≥ 1 ng/ml (*n* = 47)**	***P-*value**
Age (years)	34.2 ± 3.6	32.7 ± 3.5	0.118
Body mass index (kg/m^2^)	22.1 ± 3.2	21.4 ± 2.9	0.370
Chemotherapy regimen			0.601
AC	2 (10.0%)	3 (6.4%)	
AC followed by taxane	16 (80.0%)	35 (74.5%)	
CAF	2 (10.0%)	9 (16.4%)	
Cumulative cyclophosphamide dose, g	3,871.6 ± 528.6	3,829.3 ± 390.0	0.717
Type of GnRH agonist			1.000
Goserelin	16 (80.0%)	39 (83.0%)	
Leuplin	4 (20.0%)	8 (17.0%)	
Days from GnRH agonist administration to chemotherapy (*n*)	7.4 ± 5.8	8.2 ± 9.2	0.722
Baseline FSH	4.7 ± 2.6	4.4 ± 2.7	0.760
Baseline AMH	2.5 ± 1.5	5.5 ± 2.4	<0.001

*Data are presented as mean ± SD or n (percentage)*.

*AC, doxorubicin and cyclophosphamide; AMH, anti-Müllerian hormone; BCS, breast-conserving surgery; CAF, cyclophosphamide, doxorubicin, and 5-fluorouracil; FSH, follicle-stimulating hormone; GnRH, gonadotropin-releasing hormone*.

**Figure 2:**
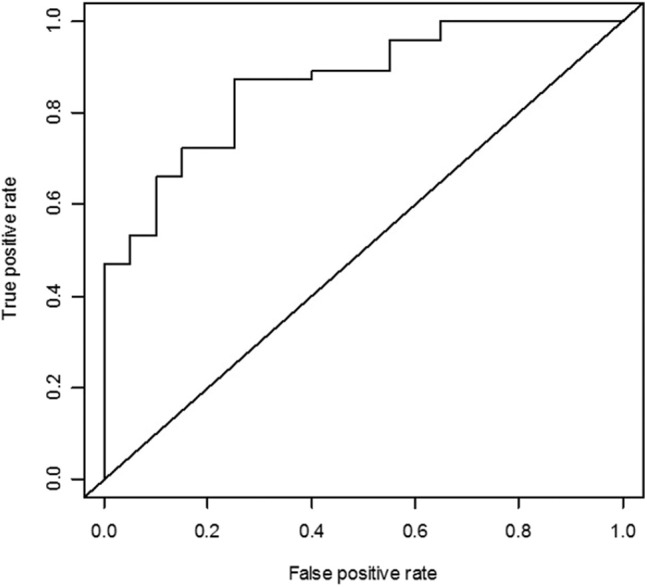
Receiver operating characteristic curve analysis of the pretreatment anti-Müllerian hormone (AMH) as the predictor for serum AMH concentration ≥1 ng/ml. The area under the curve was 0.866 for AMH ≥1 ng/ml.

From the receiver operating characteristic curve analyses of pretreatment AMH, the area under the curve was 0.866 (95% CI = 0.777–0.955) for serum AMH level ≥1 ng/ml at 12 months (Figure 2). When pretreatment AMH was considered to be the only predictive factor for successful ovarian protection, the cutoff value of pretreatment AMH for predicting AMH concentration ≥1 ng/ml at 12 months was 2.87 ng/ml. For this value, the sensitivity was 87.2%, specificity was 75.0%, positive predictive value was 89.1%, and negative predictive value was 71.4%.

## Discussion

This prospective study evaluated the factors associated with successful ovarian protection using GnRH agonists during chemotherapy in young estrogen receptor-negative breast cancer patients and demonstrated that pretreatment AMH is a useful marker for predicting the preservation of ovarian function after GnRH agonist treatment.

Age is a well-known factor associated with ovarian function after ovarian protection, but in the current study, age was not associated with successful ovarian protection at 12 months after chemotherapy. This discrepancy may result from differences in the study population among the studies—the patients in our study were much younger (mean age: 33 years) than the other studies (mean age: 38–42 years) (8–10, 19, 20) which had an upper age limit for eligibility up to 49 years. Considering that the age cutoff for higher risk of post-chemotherapy amenorrhea was 40 years (10, 21–23), the younger patients in our study were at relatively low risk for the loss of ovarian function after chemotherapy and could endure gonadotoxic chemotherapy much more. Meanwhile, the cumulative dose of cyclophosphamide was also not predictive for successful ovarian protection in the present study. This finding could also be due to the younger population in our study. However, since the desire to have a pregnancy is usually an important issue in young cancer patients, our younger study population (in their early 30s) could be considered more common candidates for fertility preservation.

Our finding is in line with previous studies showing that pretreatment AMH was a predictor of chemotherapy-related amenorrhea in breast cancer patients both without ovarian protection (24–26) as well as those with ovarian protection using GnRH agonists (10). Of note, whereas almost all women had resumed menstruation, 70% of the patients had serum AMH ≥1 ng/ml at the 12 months' follow-up in the current study. This was similar with previous studies showing that serum AMH levels decreased after chemotherapy in women who resumed menstruation (27, 28). The ~30% gap between the two parameters of ovarian function (menstruation and serum AMH) suggests that menstruation status alone is not a reliable marker for the evaluation of ovarian function after chemotherapy and that serum AMH levels must also be assessed.

Recent studies have demonstrated the efficacy of GnRH agonist treatment for ovarian protection (8–11). Temporary ovarian suppression with GnRH agonists is now recommended by several guidelines or expert opinions for fertility preservation (29–32). However, the efficacy of GnRH agonist treatment has mainly focused on preventing chemotherapy-induced amenorrhea. With regard to preserving fertility potential, there is still conflicting evidence. From many guidelines, GnRH agonists are currently not recommended as an evidence-based fertility preservation method (7) and should not preclude the established cryopreservation method. Indeed, the serum levels of AMH markedly decreased even after ovarian protection with GnRH agonists in our study and a recent study (10) even though both studies presented beneficial effects for preventing chemotherapy-related amenorrhea. In this context, our study has an important clinical impact on the consideration for fertility preservation in young breast cancer patients who require gonadotoxic chemotherapy. Cryopreservation cannot be applied to all young breast cancer patients since not every patient has enough time and money. Therefore, the identification of good candidates for only GnRH agonist treatment is very important. From the results in this study, pretreatment levels of AMH could help improve decision-making regarding fertility preservation. If pretreatment AMH levels are greater than 2.87 ng/ml, GnRH agonists can be considered more favorably. However, since cryopreservation is the only established, evidence-based fertility preservation method, such methods should be considered even in women with AMH >2.87 ng/ml.

This study has several strengths. First, the success of ovarian protection was prospectively addressed by serum AMH levels. AMH, a member of the transforming growth factor family, is produced by granulosa cells of small follicles. The AMH level is used to assess ovarian reserve, to predict ovarian response for *in vitro* fertilization, to assess damage to the ovary by surgery or chemotherapy, to predict the reproductive life span, to evaluate polycystic ovarian syndrome, and to diagnose premature ovarian insufficiency (33). In the field of oncology, AMH is useful for assessing ovarian function and advising patients before and after cancer treatment; therefore, it is promising for improving information and decision-making (34). The efficacy of GnRH agonists for ovarian protection has been criticized due to the lack of standardized diagnostic criteria. In most clinical studies assessing the use of GnRH agonist for ovarian protection, menstrual status or serum FSH and estradiol levels were used to determine successful protection. Compared to these factors, AMH levels are more objective and reliable markers for ovarian function and do not present large intra- or inter-cycle variability (32). However, the cost of testing AMH may be higher than that of FSH or estradiol. Second, compared with other studies evaluating the factors associated with amenorrhea after ovarian protection (10), more factors such as the type of GnRH agonist or days from GnRH agonist administration to chemotherapy were assessed. Finally, we only included estrogen receptor-negative breast cancer patients. In our previous study (13), both estrogen receptor-positive and -negative breast cancer patients were included for analyses, and tamoxifen use was associated with post-chemotherapy serum AMH level as well as amenorrhea. In addition, the number of estrogen receptor-negative patients was relatively small (*n* = 34). Consequently, the predictive value of AMH was different between previous and present studies. In this context, we analyzed only estrogen receptor-negative patients and demonstrated the sole effect of ovarian protection using GnRH agonists on serum AMH levels in the present study.

However, our study also has several limitations. First, the duration of follow-up was 1 year. This seems to be relatively short, considering that several studies used data over the course of 2 years. However, since the development from a surviving primordial follicle to ovulatory follicle does not take more than 6 months, 1 year would not be a short duration to evaluate the efficacy of GnRH agonists. Second, although the primary purpose of ovarian suppression is to prevent early menopause, we could not address pregnancy information due to the short follow-up period. However, we considered 1 ng/ml of serum AMH levels as successful protection, and women with diminished ovarian reserves have better delivery chances at this AMH level regardless of age (30). Finally, antral follicle count, which is a well-known surrogate marker of ovarian reserve, was not measured, and serum hormones were measured regardless of menstrual phase. This is because the present study was not interventional, and we could not delay chemotherapy only to measure antral follicle count or hormones at a specific time to reflect real clinical practice. Serum hormone levels were also measured randomly in other randomized trials.

In conclusion, pretreatment AMH is a useful marker for predicting successful ovarian protection using GnRH agonists in young estrogen receptor-negative breast cancer patients. This finding can support the decision-making process for fertility preservation.

## Data Availability Statement

The datasets generated for this study are available on request to the corresponding author.

## Ethics Statement

The studies involving human participants were reviewed and approved by Samsung Medical Center. The patients/participants provided their written informed consent to participate in this study.

## Author Contributions

D-YL, J-YK, JY, and SK were responsible for the concept and design of the study, searching for and analyzing data, and the writing of the manuscript.

## Conflict of Interest

The authors declare that the research was conducted in the absence of any commercial or financial relationships that could be construed as a potential conflict of interest.
